# Lipid Analysis of Follicular Fluids by UHPLC-ESI-HRMS Discovers Potential Biomarkers for Ovarian Hyperstimulation Syndrome

**DOI:** 10.3389/fendo.2022.895116

**Published:** 2022-06-29

**Authors:** Yue Gao, Jingjie Li, Shicheng Fan, Pan Chen, Min Huang, Huichang Bi

**Affiliations:** ^1^ Guangdong Provincial Key Laboratory of New Drug Design and Evaluation, School of Pharmaceutical Sciences, Sun Yat-Sen University, Guangzhou, China; ^2^ Center of Reproductive Medicine, The Sixth Affiliated Hospital, Sun Yat-sen University, Guangzhou, China; ^3^ Pharmacy Department, The First Affiliated Hospital, Sun Yat-sen University, Guangzhou, China; ^4^ School of Pharmaceutical Sciences, Southern Medical University, Guangzhou, China

**Keywords:** lipidomics, follicular fluids, ovarian hyperstimulation syndrome, UHPLC-ESI-HRMS, biomarkers

## Abstract

Ovarian hyperstimulation syndrome (OHSS) is a serious iatrogenic complication during ovarian stimulation. Even though the incidence of OHSS was relatively low in clinical practice, the consequence can be potentially devastating and life-threatening. Abnormal lipid metabolism may relate to the pathological development of OHSS, but there is still a research gap in the lipidomic research. So here in our study, an ultra-high-performance liquid chromatography coupled with electrospray ionization high-resolution mass spectrometry (UHPLC-ESI-HRMS) based lipidomic analysis was performed using follicular fluid samples obtained from 17 patients undergoing OHSS. The lipid profiles of OHSS patients were characterized by increased cholesterol ester (ChE) and decreased lysophosphatidylcholine (LPC), phosphatidylinositol (PI), sphingomyelin (SM), dimethylphosphatidylethanolamine (dMePE) and lysodimethylphosphatidylethanolamine (LdMePE). Totally 10 lipids including LPC(18:0), SM(d18:1/16:0), PC(18:0/18:1), PC(20:2/20:5), PC(16:0/18:1), TG(16:0/18:1/18:1), TG(16:0/18:2/18:2), TG(16:0/16:1/18:1), ChE(20:4) and TG(8:0/8:0/10:0) were selected as differential lipids. In conclusion, this study demonstrated the alteration of various lipids in OHSS patients, which suggested the key role of lipids during the development of OHSS and shed light on the further pathophysiological research of OHSS.

## Introduction

Ovarian hyperstimulation syndrome (OHSS), which occurs during controlled ovarian stimulation, is a serious iatrogenic complication of ovarian stimulation treatments for *in vitro* fertilization (IVF) (1, 2). Based on the timing of the onset of OHSS, there are early OHSS, which is defined to present 3-7 days after hCG administration, and late OHSS, which occurs 12-17 days after hCG (3). According to the severity, there are moderate and severe OHSS. Moderate OHSS is characterized by ovarian enlargement, gastrointestinal symptoms, and fluid shifts, the incidence of that is estimated to be 3% to 6%. While severe OHSS is characterized by increased vascular permeability and the shift of fluids from blood vessels to extravascular space. By contrast, the incidence of severe OHSS is much lower at about 0.2% to 1% of all IVF cycles according to a WHO report (4–7). The symptoms of severe OHSS include massively enlarged ovaries, ascites, hydrothorax, renal failure, venous embolism, and even death (8). Even though severe OHSS occurs rarely in clinical practice, the consequences can be potentially devastating (9). Currently, the pathophysiology of OHSS is not clear enough, possible casual factors include estradiol, luteinizing hormone, human chorionic gonadotropin (hCG), inflammatory mediators, the renin-angiotensin system, vascular endothelial growth factor (VEGF) and transforming growth factor-beta (TGF-β) (8, 10). For the prediction of OHSS, several risk factors have been identified, including young age (<35 years old), polycystic ovary syndrome (PCOS), prior occurrences of OHSS, increased numbers of medium- and large-sized follicles, elevated serum anti-Mullerian hormone (AMH) and estradiol (E2) levels and high levels of soluble vascular endothelial growth factor receptor 1/sFlt1 in follicular fluid (11–14). Recent studies also demonstrated that serum progesterone concentrations on the day of oocyte retrieval and cytokine levels in abdominal fluid can also be treated as predictors for OHSS (11, 15). To reduce the risk of OHSS, pharmacologic interventions are regarded as achievable and cost-effective (16). Cabergoline, metformin, intravenous (IV) calcium and the administration of albumin and hydroxyethyl starch (HES) have been reported to be effective for the prevention of OHSS (17–20).

Omics, which aims to comprehensively investigate the roles, relationships, and actions of various types of molecules in cells of an organism, includes transcriptomics, proteomics, metabolomics and epigenomics (21). Up to now, proteomics analysis has been used in OHSS studies to discover novel biomarkers. Jarkovska et al. identified a total of 19 candidate proteins differentially expressed in follicular fluid samples of OHSS compared with control group (22). Wu *et al.* reported that haptoglobin, fibrinogen and lipoprotein lipase can be served as predictive markers of OHSS in PCOS patients (23). However, there is still a research gap in the metabolomics analysis for OHSS. As a branch of metabolomics, lipidomics mainly focuses on the systematical analysis of lipids and factors that interact with lipids (24). The advances of novel analytical approaches, particularly liquid chromatography and mass spectrometry, make lipidomics an effective tool for biomedical research as well as the discovery of biomarkers and new drug targets (25). Lipids are a kind of organic molecules which are essentially formed by carbon, hydrogen, and oxygen. They possess important functions such as modulating energy reserves, forming structural features, and promoting regulatory functions. For example, triglycerides act as the main energy source of living beings; phospholipids constitute cell membranes and give them structure (26). Some kinds of lipids have strong relationship with fertilization. For instance, lipid molecules such as endocannabinoids, lysophosphatidic acid, and prostaglandins have been reported to play key roles in the process of embryo implantation in rat (27); prostaglandins may also affect endometrial receptivity.

So here in our study, we performed a lipids-targeted metabolomics analysis to investigate the pathogenesis and to explore new biomarkers for OHSS from a new perspective.

## Materials and Methods

### Study Design and Participants

46 participants enrolled at the Reproductive Medicine Center in the Sixth Affiliated Hospital of Sun Yat-sen University in China from January to July in 2019. Study protocols were approved by the University Research Ethics Board (approval number: 2017ZSLYEC-015S). Informed consent was signed by each patient recruited into this study. All the patients suffered from infertility and received IVF/ICSI. All the patients with the OHSS high-risk satisfied the following criteria: age between 20 and 35; BMI between 18 and 25 kg/m^2^; serum AMH > 1.1 ng/mL; male infertility or tubal infertility; high risk of OHSS defined as≥25 follicles ≥11 mm on hCG day (28). Exclusion criteria were polycystic ovarian syndrome (according to the Rotterdam criteria), diminished ovarian reserve, malignancy, benign ovarian cyst including endometrioma, hydrosalpinx, chromosomal abnormality. Patients enrolled in this study were grouped into OHSS or non-OHSS according to manifestation of clinical symptoms (29).

### Stimulation Protocol and Sample Collection

All patients underwent IVF or ICSI with GnRH antagonist protocol or GnRH agonist long protocol. Patients with the antagonist protocol received the GnRH antagonist cetrorelix (Cetrotide, Merck-Serono, Geneva, Switzerland) starting on the 5th day after Recombinant FSH (rFSH, Gonal-F, Serono Laboratories, Switzerland) stimulation. Triptorelin depot (IPSEN Pharma Biothech, France) was administered for down-regulation beginning in the 20st day of the cycle prior to stimulation, and rFSH administered for ovarian stimulation. The dose of gonadotropin was adjusted according to patient’s ovarian response, based on age, FSH levels, the number and size of ovarian follicles. Recombinant hCG (250 μg, Ovidrel, Merck-Serono, Geneva, Switzerland) was administrated to trigger ovulation when at least two leading follicles reached ≥18 mm in diameter.

Fluid from the leading follicles of 16-mm diameter or larger was pooled during oocyte retrieval by transvaginal ultrasound-guided aspiration (30). Follicular fluid was centrifuged at 2000 g for 15 min at room temperature. The supernatant was collected into microtubes and preserved in liquid nitrogen until analysis.

### Lipid Extraction for Lipidomic Analysis

A modified MTBE method was performed for lipid extraction (31). First, samples were thawed on ice and then 40 μL of follicular fluid was transferred to a new tube, then added with 400 μL methanol and vortexed for 30 s. Following that, 500 μL MTBE was added to the tube and vortexed for 30 s. Finally, added with 500 μL high-purity water and vortexed for another 30 s. The samples were then centrifuged at 3,000 rpm, 4°C for 10 minutes, 200 μL of supernatant from each sample was transferred to a new tube and dried by nitrogen gas. The samples were preserved at –80°C until further analysis.

For lipidomic analysis, 500 μL the mixture of methanol/isopropanol (1:1, v/v) were added to the samples and vortexed for 30 s. Then centrifuged at 18,000g, 4°C for 5 minutes. Finally, 2 μL of the supernatant was injected for ultra-high-performance liquid chromatography coupled with electrospray ionization high-resolution mass spectrometry (UHPLC-ESI-HRMS) analysis. 3 μL of the supernatant from each sample was mixed as Quality Control (QC) sample.

### UHPLC-ESI-HRMS Conditions

As described in our previous reports, the Ascentis Express C18 2.7-mm column (100 μm × 2.1 μm, Sigma-Aldrich, St.Louis, MO) was used for chromatographic separation and lipidomic analysis on a Thermo Scientific Dionex Ultimate 3000 UHPLC system (Thermo Scientific, San Jose, CA). Flow rate: 0.3 mL/min, Column Temperature: 45°C. The mobile phases were A: 5% acetonitrile in isopropanol with 10 mM ammonium formate and 0.1% formic acid. B: 50% water in acetonitrile with 10 mM ammonium formate and 0.1% formic acid. The gradient program was as follows: 20% A for 0.5 min; the proportion of A was increased to 50% at 7.5 min; then linearly increased to 80% A at 10 min; following linearly increased to 100% A at 20 min; 100% A from 20 to 21.9 min and 20% A from 22 to 25 min. Mass spectrometry was performed with a Thermo Scientific Q Exactive™ benchtop Orbitrap mass spectrometer equipped with heated ESI source in ESI positive and negative modes (Thermo Scientific). The main parameters included AGC target 1e5; maximum injection time 65 ms; apex trigger 5 to 10 seconds; dynamic exclusion 10.0 seconds; isolation window 1.2 m/z; and normalized collision energy 25 and 35 eV in positive mode and 20, 30, and 40 eV in negative mode. Ionization conditions were operated at spray voltage 3.5 kV and capillary temperature 300°C (32).

### Statistical Analysis

As described in our previous report (32), The acquired total ion chromatograms (TIC) and mass spectra from UHPLC-ESI-HRMS were exported as raw files by Xcalibur (Thermo Scientific, San Jose, CA). Lipid identification was performed by LipidSearch software (Thermo Scientific, San Jose, CA), which can also help to acquire the accurate *m/z*-value, retention time, and peak area of each metabolite. The identified lipids were evaluated by A, B, C and D four degrees, only the metabolites that were A or B levels could be selected for further data analysis. [M+H]^+^, [M+Na]^+^, and [M+NH_4_]^+^ adduct ions were considered as precursor ions in positive ion mode and [M-H]^-^ was considered as a precursor ion in negative ion mode.

Then orthogonal projection to latent structures discriminant analysis (OPLS-DA) was conducted by SIMCA-P 13.0 Software (Umetrics, Kinnelon, NJ) to visualize the difference of lipidomes in OHSS and Control group. S-plots in positive and negative modes and the variable importance in the projection (VIP) value of each lipid were acquired under OPLS-DA mode. Shapiro–Wilk test was used to evaluate the normality of distribution, then statistical significance was calculated using Student’s *t*-test and non-parametric Mann–Whitney U-test, with *p* < 0.05 as statistical significance level. Statistical test was carried out by SPSS 21.0 software (IBM Analytics, USA). The metabolites with *p* < 0.05 and VIP>1.0 were considered as the potential markers.

## Results

**Table 1** summarizes the demographic data of patients enrolled. A total of 42 patients were enrolled in the study and no patient had an embryo transfer. 17 patients were diagnosed as OHSS, stage as mild (*n*=2), moderate (*n*=13), Severe (*n*=2). Age, BMI, AMH, E_2_ and P in serum on hCG day, numbers of retrieved oocytes and total gonadotropin dosage were comparable between two groups (*p >*0.05).

**Table 1:** Characteristics of participant.

	Control (*n*=25)	OHSS (*n*=17)	*p* value
**Age (years)**	32.04 ± 0.75	30.29 ± 0.71	0.1162
**BMI (kg/m^2^)**	21.42 ± 0.43	21.43 ± 0.53	0.9804
**AMH (ng/ml)**	4.61 ± 0.52	5.88 ± 0.73	0.155
**T (ng/ml)**	0.24 ± 0.17	0.25 ± 0.125	0.926
**E2 on the day of hCG (pg/mL)**	5381 ± 323.4	5720 ± 447.6	0.5323
**P on the day of hCG (ng/mL)**	1.03 ± 0.16	0.79 ± 0.11	0.2709
**Total dose of FSH (IU)**	1972 ± 151.8	1628 ± 172.2	0.1474
**Oocytes retrieved**	17 ± 0.96	20.41 ± 1.69	0.0671

Values are presented as mean ± S.D.

**Figure 1:**
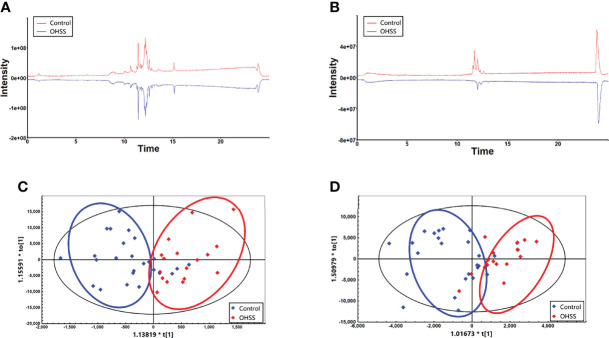
The mirror plots under positive mode **(A)** and negative mode **(B)** showed there were significant differences between Control and OHSS group. The OPLS-DA plots under positive mode **(C)** and negative mode **(D)** indicated a distinct lipid profiles between two groups.

Lipidomic analysis was then performed to explore the difference of lipidomes between OHSS and Control group. As shown in **Figures 1A, B**, the mirror plots were generated to visually observe the differences in the lipid profile. The results suggested that there were significant differences between the total ion chromatograms (TIC) in these two groups under both positive and negative mode. Additionally, OPLS-DA plots were able to clearly distinguish the OHSS and Control group under both ion modes, which indicated a distinct lipid profile between these two groups (**Figures 1C, D**).

**Figure 2:**
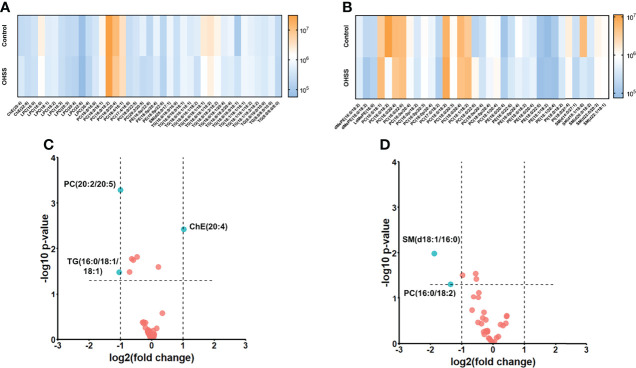
The heatmaps under positive mode **(A)** and negative mode **(B)** showed the intensities and change trend of 9 classes of lipids in Control and OHSS group. The volcano plots under positive mode **(C)** and negative mode **(D)** suggested the 5 most discriminant lipids with fold change>2.0 and *p <*0.05 (the lipids were highlighted in blue in the figures).

Totally 9 classes of lipids were identified, including cholesterol ester (ChE), lysophosphatidylcholine (LPC), phosphatidylcholine (PC), phosphatidylethanolamine (PE), sphingomyelin (SM), triacylglycerol (TG), dimethylphosphatidylethanolamine (dMePE), lysodimethylphosphatidylethanolamine (LdMePE) and phosphatidylinositol (PI). Then the heatmaps under both ion modes were obtained to visualize the change of different lipids. As shown in **Figures 2A, B**, LPC, SM, dMePE, LdMePE and PI showed higher accumulation in Control group. Most of the PE, PC and TG are lower in OHSS group while the intensity of ChE was higher in OHSS group. Then the volcano plots were generated which suggested the 5 most discriminant lipids with fold change>2.0 and *p <*0.05. (**Figures 2C, D**), including ChE(20:4), PC(20:2/20:5), TG(16:0/18:1/18:1), PC(16:0/18:2) and SM(d18:1/16:0).

**Figure 3:**
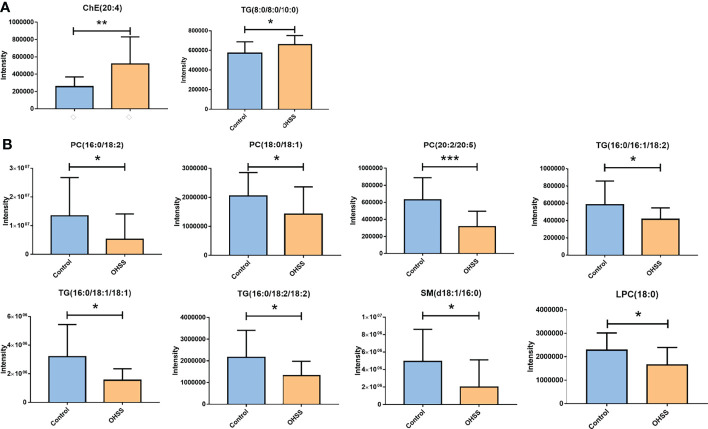
The intensities of ChE(20:4) and TG(8:0/8:0/10:0) were higher in OHSS group **(A)**. The intensities of LPC(18:0), SM(d18:1/16:0), PC(18:0/18:1), PC(20:2/20:5), PC(16:0/18:1), TG(16:0/18:1/18:1), TG(16:0/18:2/18:2) and TG(16:0/16:1/18:1) were lower in OHSS group **(B)**. Data are expressed as mean ± S.D. *p < 0.05, **p < 0.01, ***p < 0.001, OHSS patients (OHSS, *n* = 17) vs. healthy controls (Control, *n* = 25).

**Figure 4:**
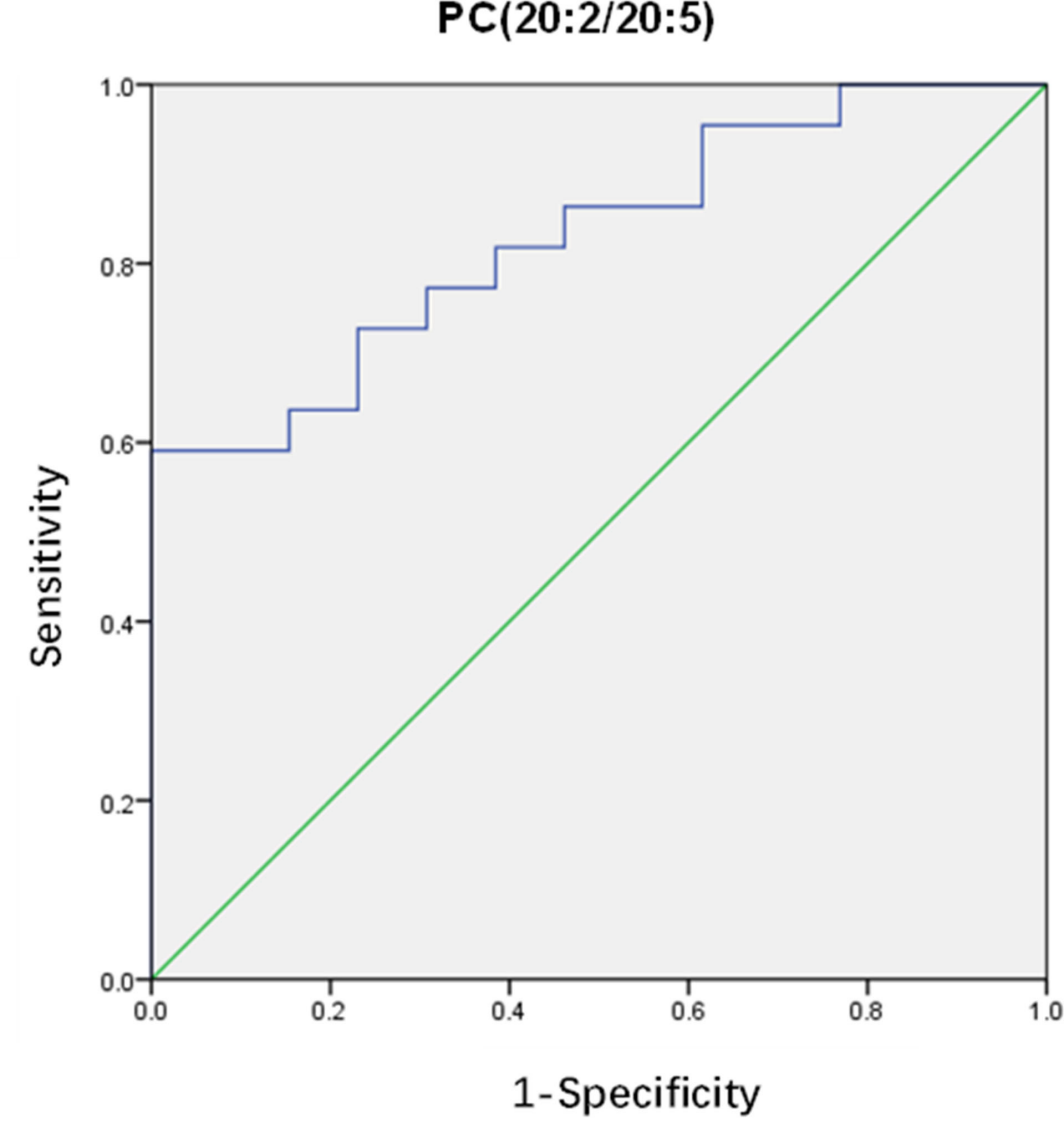
Receiver operating characteristic curve for PC(20:2/20:5).

After that, differential lipids were selected by VIP value>1.0 and *p <*0.05. Finally, 10 lipids were selected as differential lipids and the accurate *m/z*-values, retention times, and peak areas were summarized in **Table 2**. The classes of the differential lipids are mainly ChE, LPC, TG, PC and SM. Obviously, the intensities of ChE(20:4) and TG(8:0/8:0/10:0) were higher in OHSS group (**Figure 3A**), while the intensities of LPC(18:0), SM(d18:1/16:0), PC(18:0/18:1), PC(20:2/20:5), PC(16:0/18:1) and the long-chain triacylglycerols such as TG(16:0/18:1/18:1), TG(16:0/18:2/18:2) and TG(16:0/16:1/18:1) were lower in OHSS group (**Figure 3B**). Moreover, receiver operating characteristic (ROC) curves were generated, the AUC for PC(20:2/20:5) was higher than others at 0.829, with a sensitivity of 0.961 and specificity of 0.696 (**Figure 4**).

**Table 2:** Detailed information of significantly changed lipids between OHSS patients and healthy controls.

Lipid molecular	Molecular formula	Adduct	*m/z*	*p* Value	VIP Value	Fold change
**Positive mode**						
ChE(20:4)	C_47_H_76_O_2_	M+NH_4_	690.617	0.004	1.086	2.027
LPC(18:0)	C_26_H_54_O_7_N_1_P_1_	M+H	524.370	0.015	1.481	0.724
PC(18:0/18:1)	C_44_H_86_O_8_N_1_P_1_	M+H	788.613	0.017	1.423	0.641
PC(20:2/20:5)	C_48_H_82_O_8_N_1_P_1_	M+H	832.582	0.0005	1.780	0.499
TG(16:0/16:1/18:2)	C_53_H_96_O_6_	M+NH_4_	846.753	0.018	1.466	0.664
TG(16:0/18:1/18:1)	C_55_H_102_O_6_	M+NH_4_	876.799	0.033	1.693	0.485
TG(16:0/18:2/18:2)	C_55_H_98_O_6_	M+NH_4_	872.769	0.033	1.829	0.610
TG(8:0/8:0/10:0)	C_29_H_54_O_6_	M+NH_4_	516.425	0.025	1.275	1.153
**Negative mode**						
PC(16:0/18:2)	C_42_H_80_O_8_N_1_P_1_	M+HCOO	802.561	0.0495	4.008	0.391
SM(d18:1/16:0)	C_39_H_79_O_6_N_2_P_1_	M+HCOO	747.566	0.010	1.501	0.273

## Discussions

OHSS is a serious iatrogenic complication of ovarian stimulation treatments for *in vitro* fertilization, however, the pathophysiology of this disease is not clear enough. Lipids possess various function in cells and have been reported to play important roles in various diseases (27), but the alteration of lipids in OHSS patients has not been systematically investigated. In our current study, an UHPLC-ESI-HRMS based lipidomic analysis was conducted, totally 9 classes of lipids were identified and we found that LPC, SM, dMePE, LdMePE, PI and most of the PE, PC and TG are lower in OHSS group while the intensity of ChE was higher in OHSS group. According to the selection standard, 10 lipids including LPC(18:0), SM(d18:1/16:0), PC(18:0/18:1), PC(20:2/20:5), PC(16:0/18:1), TG(16:0/18:1/18:1), TG(16:0/18:2/18:2), TG(16:0/16:1/18:1), ChE(20:4) and TG(8:0/8:0/10:0) were selected as differential lipids and might be served as biomarkers in the future.

The follicular fluid samples obtained from 17 patients were used in our study for lipidomic analysis. Follicular fluid (FF) is a liquid constituted with hormones, enzymes, anticoagulants, electrolytes, reactive oxygen species and antioxidants, it fills the follicular antrum and acts as an important mediator between cells in the antral follicle while bathing and carrying nutrients to the oocyte (33). FF is an important pool for biomarkers to predict the quality of oocytes and embryos (34), which can be served as ideal samples for non-invasive diagnosis. The levels of cytokines, extracellular microRNAs and melatonin in follicular fluid can be used as markers for IVF outcomes (35–37). In the current research, the FF samples from OHSS patients were collected due to the key role of FF in fertilization, the differential lipids reported in our study might be served as potential biomarkers and these results also provided a new prospect for the mechanism study of OHSS in the future. Blood samples were not used for lipidomic analysis in our research, blood-derived serum and plasma are the sample matrices most influenced by various factors including fasting time, handling procedures and other metabolic diseases, which may reduce the reliability of the results. What’s more, the lipid profiles also differ between serum and plasma, it is difficult to state which type of blood-based sample is optimal for lipidomic analysis (38). FF provides the microenvironment for the oocyte development, the lipid contents are relatively stable compared with blood samples. Lipidomics analysis for FF may give more valuable and accurate information regarding potential biomarkers of OHSS (39). However, to identify the biomarkers which can be applied in clinal practice, the data from FF and the data from blood samples should be collected simultaneously for correlation analysis.

Lipidomics is now an effective tool to investigate lipids metabolism-related diseases. A systematic lipidomic analysis for OHSS was carried out in the current study and we performed that on the basis of two previous reports. On the one hand, Liu et al. reported that dyslipidemia might contribute to the development of OHSS, which suggested that abnormal lipid metabolism might be a risk factor for OHSS (40). On the other hand, a proteomic analysis for OHSS in PCOS patients suggested that lipoprotein lipase, a protein which catalyses the hydrolysis of the triacylglycerol component of circulating chylomicrons and very low-density lipoproteins to provide non-esterified fatty acids and 2-monoacylglycerol for tissue utilization (23, 41), can be served as a predictive marker of OHSS in PCOS patients. Based on these researches, we hypothesized that lipid metabolism might have strong relationship with the occurrence of OHSS, so we performed this study to investigate the changes of lipids in OHSS patients.

According to the results, ChE was higher in OHSS group. It has been reported that cholesteryl ester transfer protein (CETP), a hydrophobic glycoprotein transfers cholesterol from the cytoplasm to the outside of the cell *via* the cell membrane, plays a key role in the maintenance of vesicle membrane integrity (42). The elevation of ChE in OHSS patients may reflect the change of CETP in FF and further affect the vascular permeability to induce OHSS.

Elevated TG(8:0/8:0/10:0) can be observed in OHSS patients in the current study. It has been illustrated that inflammation is associated with the occurrence of OHSS. Gonzalez et al. demonstrated that inflammatory markers including pro-inflammatory (CRP, sICAM-1, IL6, TNFα) and anti-inflammatory (adiponectin, IL10) are correlated with follicular triglycerides (43). Particularly, sICAM-1 may be affected by mode of ovarian stimulation and even reflect OHSS. However, the levels of TG(16:0/18:1/18:1), TG(16:0/18:2/18:2) were lower in OHSS group, we hypothesize that the different changing trend of theses TGs may due to the length of carbon chain and the number of unsaturated bonds. Further investigations are needed to clarify this phenomenon.

Phospholipids (PLs) such as LPC, dMePE, LdMePE, PI and most of the PE, PC were downregulated in OHSS patients. PLs are essential components of cell membranes which are ubiquitous to all tissues. The main function of PLs is to support the formation and maintain the function of cell membranes, there are specific varied PLs that perform specialized functions in the subcellular micelles and organelles. Some PLs also act as mediators of inflammation to influence immunological processes at the cellular level. Therefore, PLs not only participate in the formation of cell membranes, but also act as messengers in cell signaling (44). Additionally, oxidized phospholipids (OxPLs), which exert distinct biological effects, are well recognized to contribute to the induction and resolution of inflammation. We inferred that the change of PLs might associate with inflammation (45). Further studies are needed to explore the change of OxPLs and their relationship with OHSS.

SM is a kind of lipids which consists of sphingosine and bares a choline molecule. It is found in high quantities in brain and neural tissues membranes (44). Cordeiro *et al.* investigated follicular metabolic characteristics associated with hyper response to COS, the results indicated that SM is relatively abundant in Control group, which were in consistent with our results (46). Overall, sphingolipids are associated with the synthesis of steroid hormones, mainly through the modulation of steroidogenic pathways by sphingosine-1-phosphate (S1P), which has been elucidated to affect ovarian capillary permeability and the expression of VEGF_121_ isoform and its receptor KDR in the ovaries from an OHSS rat model (46, 47).

## Conclusion

In conclusion, the current study demonstrated the alteration of lipids in FF samples from OHSS patients through lipidomic analysis. An elevation of ChE and a decrease of LPC, SM, dMePE, LdMePE, PI and most of the PE, PC and TG can be observed in OHSS group. The lipid PC(20:2/20:5) showed a high AUC, as well as sensitivity and specificity, which might be used as a biomarker in clinical practice in the future. These findings provided a new prospect for the pathophysiological studies and potential biomarkers identification of OHSS. However, further studies are needed to investigate the significance and importance of these differential lipids in OHSS.

## Data Availability Statement

The original contributions presented in the study are included in the article/supplementary material. Further inquiries can be directed to the corresponding author.

## Ethics Statement

The studies involving human participants were reviewed and approved by University Research Ethics Board. The patients/participants provided their written informed consent to participate in this study.

## Author Contributions

JL and HB conceived and designed the study. JL and PC collected the samples. YG and SF performed the experiments and analyzed the data. YG and JL wrote the manuscript. MH and HB revised the manuscript. All authors contributed to the article and approved the submitted version.

## Funding

The work was supported by the Natural Science Foundation of China (Grants: 82025034, 81973392, 82000593); the National Key Research and Development Program (Grant: 2017YFE0109900); the Shenzhen Science and Technology Program (No. KQTD20190929174023858); the Natural Science Foundation of Guangdong (Grant: 2017A030311018); the 111 project (Grant: B16047); the Key Laboratory Foundation of Guangdong Province (Grant: 2017B030314030); the Local Innovative and Research Teams Project of Guangdong Pearl River Talents Program (2017BT01Y093); and the National Engineering and Technology Research Center for New drug Druggability Evaluation (Seed Program of Guangdong Province, 2017B090903004).

## Conflict of Interest

The authors declare that the research was conducted in the absence of any commercial or financial relationships that could be construed as a potential conflict of interest.

## Publisher’s Note

All claims expressed in this article are solely those of the authors and do not necessarily represent those of their affiliated organizations, or those of the publisher, the editors and the reviewers. Any product that may be evaluated in this article, or claim that may be made by its manufacturer, is not guaranteed or endorsed by the publisher.
